# Role of Src in Vascular Hyperpermeability Induced by Advanced Glycation End Products

**DOI:** 10.1038/srep14090

**Published:** 2015-09-18

**Authors:** Weijin Zhang, Qiulin Xu, Jie Wu, Xiaoyan Zhou, Jie Weng, Jing Xu, Weiju Wang, Qiaobing Huang, Xiaohua Guo

**Affiliations:** 1 Department of Pathophysiology, Key Laboratory for Shock and Microcirculation Research of Guangdong Province, Southern Medical University, Guangzhou 510515, China; 2 Department of Intensive Care Unit, General Hospital of Guangzhou Military Command, Guangzhou, 510010, China; 3 Postdoctoral Workstation, Huabo Bio-pharmaceutical Research Institute, Guangzhou 510515, China

## Abstract

The disruption of microvascular barrier in response to advanced glycation end products (AGEs) stimulation contributes to vasculopathy associated with diabetes mellitus. Here, to study the role of Src and its association with moesin, VE-cadherin and focal adhesion kinase (FAK) in AGE-induced vascular hyperpermeability, we verified that AGE induced phosphorylation of Src, causing increased permeability in HUVECs. Cells over-expressed Src displayed a higher permeability after AGE treatment, accompanied with more obvious F-actin rearrangement. Activation of Src with pcDNA3/flag-Src^Y530F^ alone duplicated these effects. Inhibition of Src with siRNA, PP2 or pcDNA3/flag-Src^K298M^ abolished these effects. The pulmonary microvascular endothelial cells (PMVECs) isolated from receptor for AGEs (RAGE)-knockout mice decreased the phosphorylation of Src and attenuated the barrier dysfunction after AGE-treatment. *In vivo* study showed that the exudation of dextran from mesenteric venules was increased in AGE-treated mouse. This was attenuated in RAGE knockout or PP2-pretreated mice. Up-regulation of Src activity induced the phosphorylation of moesin, as well as activation and dissociation of VE-cadherin, while down-regulation of Src abolished these effects. FAK was also proved to interact with Src in HUVECs stimulated with AGEs. Our studies demonstrated that Src plays a critical role in AGE-induced microvascular hyperpermeability by phosphorylating moesin, VE-cadherin, and FAK respectively.

Microvascular barrier dysfunction and endothelial hyperpermeability are the crucial events in the development of inflammatory diseases, such as trauma, ischemia-reperfusion injury, arteriosclerosis, and especially, diabetes mellitus (DM). Vascular endothelial cells lining the intima of the blood vessels to form a semi-permeable barrier are the bases of microvascular barrier function. The disrupted barrier in response to a variety of stimuli causes endothelial hyperpermeability, exudation of vascular contents and inflammatory factors, transmigration of inflammatory cells, resulting in tissue edema and organ dysfunction. Advanced glycation end products (AGEs) are a group of compounds produced by the non-enzymatic glycation or glycoxidation of proteins, lipids, and nucleic acids, and play a crucial role in the pathogenesis of diabetic microangiopathy and macrovasculopathy. It is reported that AGEs accumulate in the tissue and plasma during aging, while markedly increase in patients with diabetes^1^. Numerous studies have showed that AGEs are associated with the generation of reactive oxygen species (ROS), impaired anti-oxidative functions of high density lipoprotein (HDL), and increased inflammatory cytokines^2^,^3^,^4^. We and others have reported that AGEs are implicated in microvascular barrier dysfunction and endothelial hyperpermeability in DM^5^,^6^,^7^. Although study focused on the development of anti-AGE agent failed to show significant benefit in clinical trials^8^, the therapy targeting AGEs and its signaling pathway remains a hot area of research in DM. Therefore, better understanding of the exact mechanisms underlying diabetic vascular diseases could provide a feasible preventive strategy and promising therapeutic approach for the vascular complication of DM.

Src family kinases (SFKs) are the largest family of non-receptor tyrosine kinase, consisting of nine structurally related proteins, Src, Blk, Fyn, Yes, Lyn, Lck, Hck, Fgr and Yrk. These proteins share four Src homology (SH) domains involved in catalytic activity, protein-protein interaction, and cell membrane binding^9^. SFKs are maintained at an inactivate state by the interaction between SH2 and the phosphorylated C-terminal tyrosine, Tyr530. And dephosphorylation at Tyr530 by multiple phosphatases can switch them from inactive to active state. Mutations at Tyr530 lead to constitutive enzymatic activity, while at Lys298, the active site of enzyme, cause catalytic deficiency^9^,^10^. SFKs play an important role in proliferation, apoptosis, cell cycle control, angiogenesis, and cell-cell adhesion and communication. Recent studies showed that SFK signaling is important in the regulation of microvascular barrier function and various endothelial responses to a diversity of inflammatory mediators^11^,^12^. The main underlying mechanisms involved are as follows: (1) SFKs regulate the phosphorylation of proteins that promote cytoskeleton contraction^13^; (2) SFKs affect junctional complex by the phosphorylation of vascular endothelial cadherin (VE-cadherin), which results in the disruption of cadherin-actin complex and endothelial hyperpermeability; (3) SFKs affect vascular permeability through the regulation of focal adhesion complexes which contain integrins, focal adhesion kinase (FAK), and multiple adaptor proteins. The Src family kinases (SFKs) c-Src and Yes mediate vascular leakage in response to various stimuli including lipopolysaccharide (LPS) and vascular endothelial growth factor (VEGF). On the contrary, Lyn strengthens endothelial junctions and thereby restrains the increase in vascular permeability^14^. However, the exact role of a given SFK and the signal pathway remain unclear and seem to depend on the context and the type of stimuli.

Src is one of the most widely studied members in SFKs. Inhibition of Src has been reported to abolish the increases in albumin permeability caused by C5a-activated neutrophils, which indicates its significance in vascular hyper-permeability^15^. However, Src-directed VE-cadherin phosphorylation appears insufficient to disrupt endothelial barrier given that the dominant negative C-terminal Src kinase (Csk) and Csk knockdown did induce VE-cadherin phosphorylation, but did not cause the loss of endothelial barrier function^16^, which suggested that activation of other signals is concurrently required. ERM protein is the cross linker between plasma membrane and actin filaments. The activation of ERM is engaged in regulating cellular contraction, cell motility, microvilli formation, cell adhesion, etc. Src family kinases also mediate the phosphorylation of ERM protein, and Src specific inhibitor PP2 inhibits the activation of ERM, while moesin is the main ERM molecule expressed by endothelium. In this study, we demonstrated Src acted as a signaling node which transduced the signal of AGEs on the ligation of RAGE to moesin, VE-cadherin, and FAK, resulting in the disruption of endothelial barrier and the increase of vascular permeability.

## Results

### Effect of AGEs on endothelial monolayer permeability

**Figure 1:**
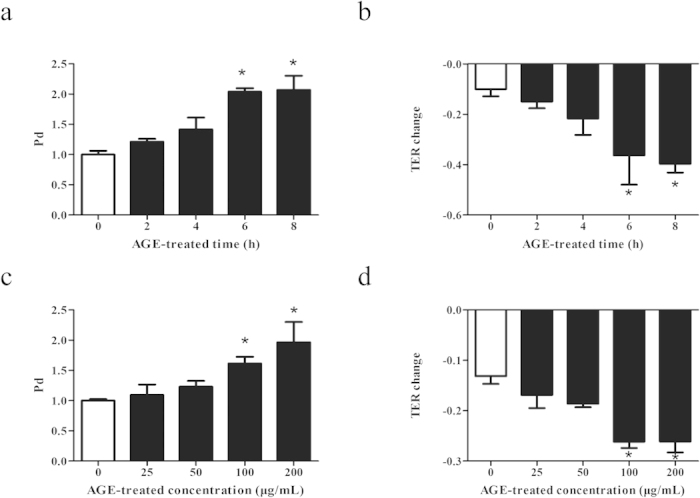
Influences of AGEs on endothelial permeability. HUVECs were either (**a**,**b**) treated with100 μg/mL of AGEs for 2, 4, 6, or 8 h, or (**c**,**d**) stimulated with AGEs at 25, 50, 100, or 200 μg/mL for 8 h, and those incubated with culture medium were used as control. Permeability coefficient of the transflux of tracer FITC-dextran (Pd) and trans-endothelial electronic resistance (TER) were measured. n = 3, *P < 0.05 versus control.

We have previously reported that AGEs exerted dose- and time-dependent effects on monolayer permeability of human dermal microvascular endothelial (HMVECs)^7^. Here, human umbilical vein endothelial cells (HUVECs) representing venous endothelial cells were used and the concentration- and time-dependent effects of AGE modified bovine serum albumin (AGE-BSA) on monolayer permeability were explored. Firstly, HUVECs were treated with100 μg/mL of AGE-BSA for different duration, and endothelial monolayer permeability was evaluated by dextran trans-endothelial flux using permeability coefficient for dextran (Pd) and trans-endothelial electric resistance (TER). We found that Pd was gradually increased from 2 h to 8 h and the changes became significant at 6 h and 8 h (Fig. 1a). Accordingly, TER was gradually decreased with significant differences at 6 h and 8 h (Fig. 1b). These data indicated a time-dependent increase in endothelial permeability induced by AGE-BSA. When cells were incubated with different concentrations of AGE-BSA for 8 h, Pd was increased and TER was decreased gradually, with significant differences at 100 μg/mL and 200 μg/mL (Fig. 1c,d), indicating AGE-BSA induced endothelial hyperpermeability in a dose-dependent fashion. These data were important since the generation of AGEs was increased with aging and elevating of blood glucose.

### Role of Src in AGE-induced endothelial hyperpermeability

**Figure 2:**
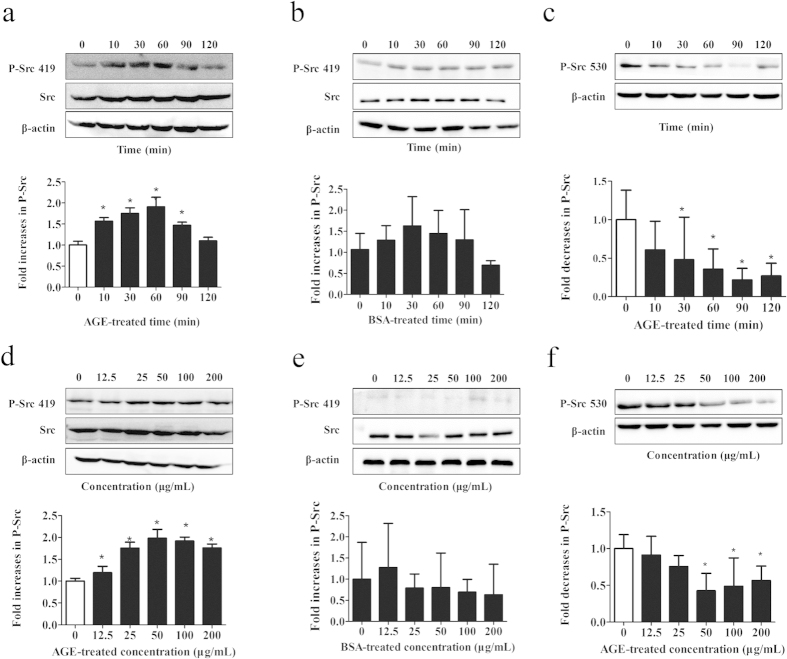
Effects of AGEs on Src phosphorylation. HUVECs were either (**a**,**c**) incubated with 100 μg/mL of AGEs for 10, 30, 60, 90, or 120 min, or (**d**,**f**) stimulated with 12.5, 25, 50, 100, or 200 μg/mL AGEs for 60 min. Specific antibody was used to detect P-Src 419 (**a**,**d**) and P-Src 530 (**c**,**f**) by western blotting. The effect of BSA alone on Src 419 phosphorylation was also detected as BSA control by incubating the cells with (**b**) 100 μg/mL of BSA for 10, 30, 60, 90, 120 min, or with (**e**) 12.5, 25, 50, 100, 200 μg/mL BSA for 60 min. Those incubated with culture medium were used as blank control. The ratio of immunointensity between the phosphorylation of Src (p-Src 419, p-Src 530) and total Src were calculated. n = 3, *P < 0.05 versus control.

Once activated through phosphorylation at Tyr 419, the tyrosine kinase of Src can phosphorylate many proteins involved in the regulation of endothelial permeability. We asked whether Src activation was involved in the signal events evoked by AGEs in HUVECs. Cells were incubated with 100 μg/mL of AGE-BSA for different duration and phosphorylation of Src was determined by western blotting. The results showed that phosphorylation of Src 419 was rapidly enhanced at 10 min, reached a peak at 60 min, and then returned to baseline level at 120 min, while phosphorylation of Src 530 showed the contrary tendency during 120 min period. Incubation with BSA (Bovine Serum Albumin) alone had no effect on Src 419 phosphorylation (Fig. 2a–c). Src activation was then examined in HUVECs stimulated with different concentrations of AGE-BSA for 60 min. We found that Src 419 phosphorylation was significantly increased by AGE-BSA at the concentrations ranging from 25 μg/mL to 200 μg/mL, with the peak from 50 to 100 μg/mL. On the contrary, Src 530 phosphorylation was decreased by AGE-BSA at the same concentrations range, with the peak at 50 μg/mL. Different doses of BSA alone showed no significant effects on phosphorylation of both Src 419 and Src 530 (Fig. 2d–f). These data strongly suggest that Src activation was linked with AGE-induced disruption of endothelial barrier. We also noticed that, compared to 100 μg/mL, the endothelial monolayer permeability increases further at 200 μg/mL, but Src phosphorylation decreases at 200 μg/mL relative to 100 μg/mL. It is possible that some other signal pathways, not only Src, might participate in high dose AGE-induced hyperpermeability, which needs to be investigated in the future.

**Figure 3:**
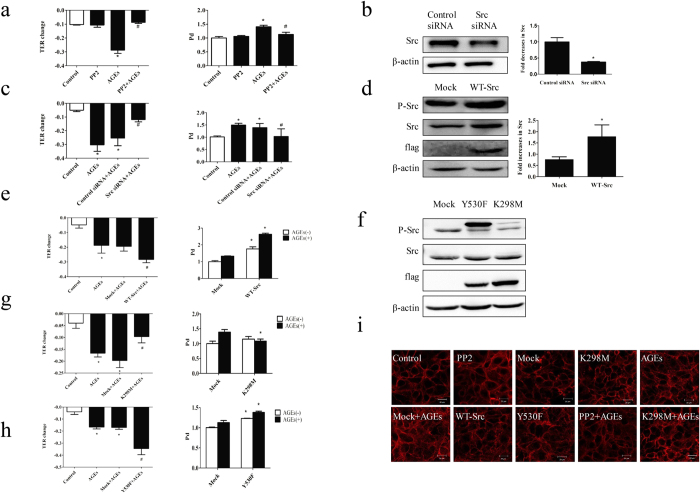
Influences of Src signaling on endothelial permeability. (**a**) PP2 prevented AGE-induced hyperpermeability. HUVECs were treated with PP2 90 min before AGEs (100 μg/mL, 8 h) application. Culture medium was used as control. n = 3 *P < 0.05 versus control, ^#^*P* < 0.05 versus AGEs. (**b**) Src siRNA caused reduction in Src protein level compared with control siRNA. HUVECs were transfected with Src siRNA and control siRNA for 48 h, after which cells were lysed and detected for Src protein level by WB. (**c**) Src siRNA prevented AGE-induced hyper-permeability. HUVECs were transfected with siRNA for 48 h before exposure to AGEs (100 μg/mL) for 8 h. n = 3, *P < 0.05 versus control, ^#^*P* < 0.05 versus control siRNA with AGEs. (**d**,**f**) The effects of pcDNA3/flag-Src (WT-Src), K298M and Y530F on expression of Src were detected by western blotting. (**e**,**g**,**h**) The effects of WT-Src, K298M and Y530F on EC permeability. Cells were transfected with WT-Src, K298M and Y530F with or without stimulation of 100 μg/mL AGEs for 8 h. TER and Pd were detected. n = 3, *P < 0.05 versus control or mock, ^#^*P* < 0.05 versus AGEs or mock with AGEs. (**i**) The effects of Src on the distribution of F-actin induced by AGEs. ECs were treated with or without 100 μg/mL AGEs for 8 h, followed by rhodamine-phalloidin staining, and F-actin was revealed by a laser confocal microscopy.

To further clarify the role of Src in AGE-induced endothelial barrier disruption, HUVECs were pretreated with a pan inhibitor of Src, PP2, for 90 min before stimulation with AGE-BSA. We revealed that PP2 significantly attenuated AGE-induced endothelial hyperpermeability (Fig. 3a), indicating that Src was involved in endothelial hyperpermeability induced by AGEs. To confirm this role of Src, cells were transfected with Src siRNA or its overexpression vector (WT-Src) before AGE-BSA stimulation. Knockdown of Src significantly decreased AGE-induced endothelial hyperpermeability, while Src overexpression obviously increased it (Fig. 3b,c). Moreover, Src overexpression alone increased endothelial permeability in cells without AGEs stimulation (Fig. 3d,e). To clarify whether the tyrosine kinase activity of Src was linked with AGE-induced endothelial hyperpermeability, we constructed a kinase-deficient mutant at Lys298 (K298M) and a constitutive active mutant at Tyr530 (Y530F) (Fig. 3f). We found that K298M did not change endothelial permeability in the absence of AGE-BSA, while K298M completely abolished endothelial hyperpermeability induced by AGEs (Fig. 3g). On the contrary, Y530F increased endothelial permeability in the absence of AGE-BSA, and further increased it in the presence of AGEs (Fig. 3h). These data suggest that Src activation mediated AGE-induced endothelial hyperpermeability and it was the tyrosine kinase activity of Src that triggered the downstream signaling events.

### Influences of Src on F-actin rearrangement

To investigate whether Src activation influence the cytoskeleton arrangement, F-actin morphology was revealed by rhodamine-phalloidin staining (Fig. 3i). We found that F-actin was mainly localized in the periphery of cell with continuous intact lines and typical cobblestone outline in control HUVECs. AGE treatment caused a redistribution of F-actin in the cytoplasm with dense and rough outline, and irregular jagged-like fracture. These morphologic changes became more significant in cells transfected with WT-Src or activated Src Y530F, while the AGE-induced F-actin rearrangement were inhibited by PP2 and inactivated Src K298M.

### AGE-induced phosphorylation of Src and endothelial hyperpermeability requires RAGE

**Figure 4:**
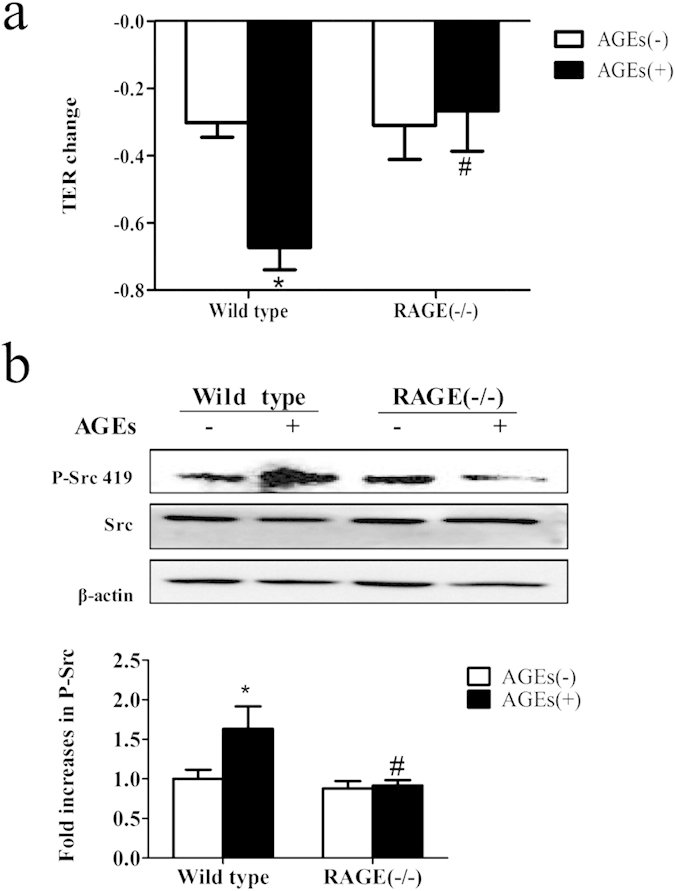
Involvement of RAGE in AGE-induced phosphorylation of Src and hyperpermeability. (**a**) Knockout of RAGE prevented AGE-induced PMVEC hyper-permeability. PMVECs of wild type mice and RAGE knockout mice were treated with or without 100 μg/mL AGEs for 90 min and TER was measured. (**b**) Knockout of RAGE prevented AGE-induced Src phosphorylation. PMVECs from wild type and RAGE knockout mice were treated with or without 100 μg/mL AGEs for 1 h. Phosphorylation of Src was detected by western blotting. The ratio of immunointensity between the phosphorylation of Src (p-Src 419) and β-actin were calculated. n = 3, *P < 0.05 versus wild type without AGEs, ^#^*P* < 0.05 versus wild type with AGEs.

To investigate whether RAGE is required for Src activation in AGE-induced endothelial dysfunction, pulmonary microvascular endothelial cells (PMVECs) isolated from wild type and RAGE knockout mice were used. Our data showed that the elevated endothelial permeability by AGEs was completely abolished by RAGE knockout (Fig. 4a). Moreover, AGE-induced Src phosphorylation was inhibited in RAGE-knockout cells (Fig. 4b). These results suggest that AGE-evoked Src activation and consequent endothelial hyperpermeability was through the ligation of RAGE.

### AGEs-mediated venular hyperpermeability is inhibited by PP2 and RAGE knockout

**Figure 5:**
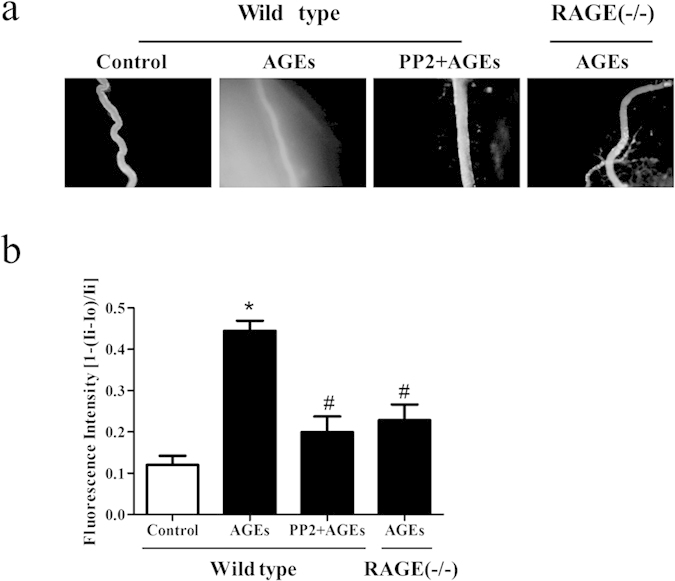
PP2 and knockout of RAGE prevents AGE-induced microvascular hyperpermeability. (**a**,**b**) Wild type (WT) or RAGE^−/−^ mice were pretreated with AGEs or AGEs plus PP2, The exudation of FITC-dextran from mesenteric venules was determined. Vascular permeability is expressed as the relative fluorescent intensity inside the vessel to that outside the vessel. n = 3, *P < 0.05 versus control group, ^#^*P* < 0.05 versus AGEs. Each image was recorded at a 100 × magnification.

The role of Src and RAGE in microvascular permeability was investigated in AGE-treated wild type (WT) and RAGE knockout mice. We revealed no exudation of FITC-dextran out of the venules in WT mice without AGE treatment. AGE-treated WT mice showed a significant increase in the exudation of FITC-dextran in the extravascular space. This exudation was markedly reduced by a pan Src inhibitor, PP2, and completely abated in RAGE knockout mice (Fig. 5). These findings suggest that Src and RAGE played an important role in AGE-induced microvascular hyperpermeability.

### Role of Src in AGE-induced phosphorylation of moesin

**Figure 6:**
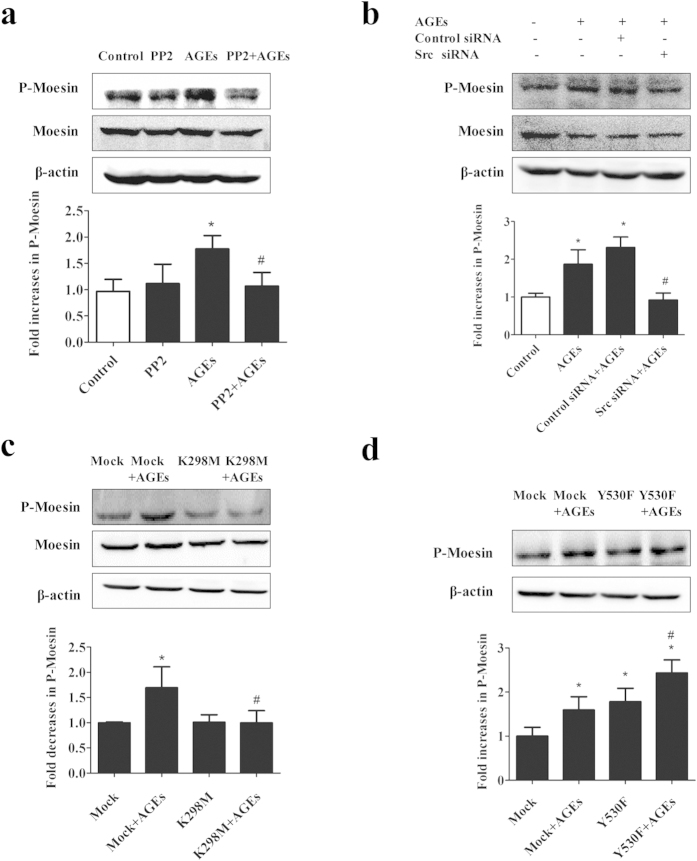
AGE-induced moesin activation requires Src. (**a**) Pretreatment of PP2 prevented AGE-induced moesin phosphorylation. ECs were pretreated with PP2 (15 μmol/L) for 90 min before exposed to 100 μg/mL AGEs for 1 h. (**b**) Pretreatment of Src siRNA prevented AGE-induced moesin phosphorylation. ECs were pretreated with Src siRNA or control siRNA for 48 h before exposed to 100 μg/mL AGEs for 1 h. (**c**) Effects of K298M and Y530F on AGE-induced moesin phosphorylation. ECs were transfected with K298M and Y530F for 48 h before exposed to 100 μg/mL AGEs for 1 h. Moesin and phosphorylated moesin were determined by western blotting. n = 3, *P < 0.05 versus control, ^#^*P* < 0.05 versus AGEs or Mock + AGEs.

Since moesin regulates endothelial permeability via modulating equilibrium between contractile forces generated by the endothelial cytoskeleton and adhesive forces produced at inter-endothelial junctions and cell-matrix attachment, here we tried to find out whether Src signaling influenced moesin phosphorylation. Consistent with our previous findings^7^, AGEs induced a significant increase in moesin phosphorylation. However, this increase was abolished by Src inhibitor PP2 or its siRNA (Fig. 6a,b). We also observed that AGE-induced moesin phosphorylation was decreased by K298M, while further increased by Y530F (Fig. 6c,d). These data suggested that Src signaling mediated AGE-induced moesin phosphorylation.

### Role of Src in AGE-induced VE-cadherin

**Figure 7:**
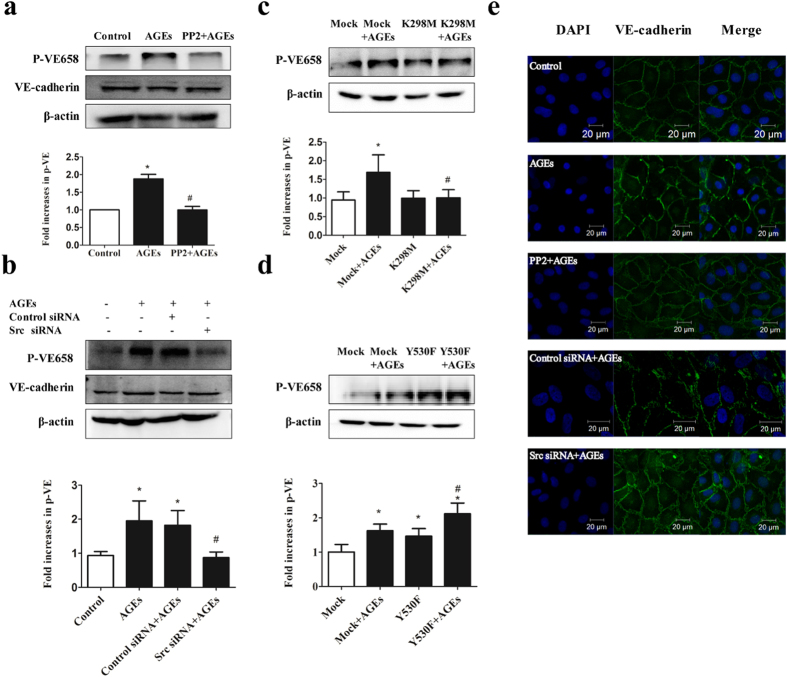
AGE-induced VE-cadherin activation requires Src. (**a**) Pretreatment of PP2 prevented AGE-induced VE-cadherin phosphorylation. ECs were pretreated with PP2 (15 μmol/L) for 90 min before exposed to 100 μg/mL AGEs for 1 h. (**b**) Pretreatment of Src siRNA prevented AGE-induced VE-cadherin phosphorylation. ECs were transfected with Src siRNA or control siRNA for 48 h before exposed to 100 μg/mL AGEs for 1 h. (**c**,**d**) Effects of K298M and Y530F on AGE-induced VE-cadherin phosphorylation. ECs were transfected with K298M or Y530F for 48 h before exposed to 100 μg/mL AGEs for 1 h. VE-cadherin and its phosphorylation form were determined by western blotting. The ratio of immunointensity between the phosphorylation of VE-cadherin (p-VE658) and β-actin were calculated. n = 3, *P < 0.05 versus control, ^#^*P* < 0.05 versus AGEs or Mock + AGEs. (**e**) Down-regulation of Src prevented AGE-induced adherence junction dissociation. HUVECs were pretreated with PP2 (15 μmol/L) for 90 min or transfected with Src siRNA for 48 h, followed by incubation with or without 100 μg/mL AGEs for 8 h. Immunostaining for VE-cadherin (green) and DAPI (blue) were shown.

Given that Src acted as an upstream of VE-cadherin and caused the disruption of endothelial barrier in response to permeability-increasing agents such as bradykinin and histamine^17^, we investigated whether Src influenced the distribution of VE-cadherin under AGE treatment. We revealed that AGEs induced the dissociation of VE-cadherin, which was prevented by inhibition of Src signaling with PP2 or Src siRNA (Fig. 7e). We also assessed the effects of Src on the phosphorylation of VE-cadherin. Our data showed that AGEs promoted VE-cadherin phosphorylation, which was blocked by PP2, Src siRNA (Fig. 7a,b), or K298M, but significantly increased by Y530F (Fig. 7c,d). Moreover, cells transfected with Y530F displayed a higher level of VE-cadherin phosphorylation compared with mock-transfected cells (Fig. 7d). These data suggest Src played a role in AGE-induced VE-cadherin phosphorylation and dissociation.

### AGE-induced FAK activation requires Src

**Figure 8:**
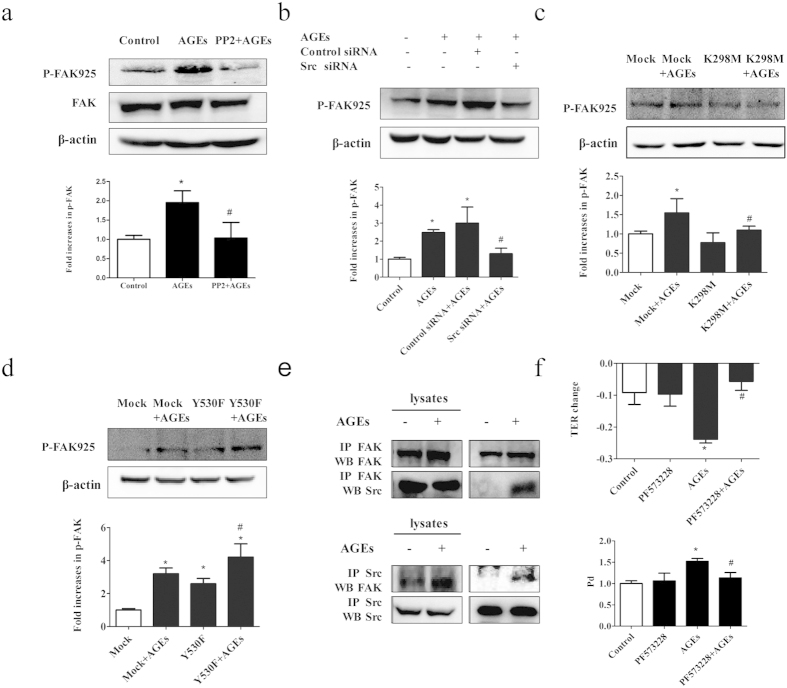
AGE-induced FAK activation requires Src. (**a**) Pretreatment of PP2 prevented AGE-induced FAK phosphorylation. ECs were pretreated with PP2 (15 μmol/L) for 90 min before exposed to 100 μg/mL AGEs for 1 h. (**b**) Pretreatment of Src siRNA prevented AGE-induced FAK phosphorylation. ECs were pretreated with Src siRNA or control siRNA for 48 h before exposed to 100 μg/mL AGEs for 1 h. (**c**,**d**) Effects of K298M and Y530F on AGE-induced FAK phosphorylation. ECs were pretreated with K298M and Y530F for 48 h before exposed to 100 μg/mL AGEs for 1 h. FAK and its phosphorylation form were detected by western blotting. (**e**) AGE induced Src/FAK association in HUVECs. HUVECs were treated with 100 μg/mL AGEs for 1 h and lysed, and co-immunoprecipitation (co-IP) for FAK was performed. Increased expression of Src was detected by western blotting (upper panel). HUVECs were treated with 100 μg/mL AGEs for 1 h and co-IP for Src was performed. Increased expression of FAK was detected by western blotting (lower panel). (**f**) PF573228 prevented AGE-induced hyper-permeability of endothelial cells. HUVECs were treated with PF573228 (20 μmol/L) for 60 min before exposed to 100 μg/mL AGEs for 8 h. Culture medium was used as control. Permeability coefficient of the transflux of trace FITC-dextran (Pd) and TER were measured. The ratio of immunointensity between the phosphorylation of FAK (p-FAK 925) and β-actin were calculated. n = 3, *P < 0.05 versus control, ^#^P < 0.05 versus AGEs group or Mock + AGEs.

To explore whether FAK was the downstream target of Src in AGE-treated HUVECs, cells were transfected with Src siRNA or pretreated with PP2 before the application of AGE-BSA. We found that AGE-BSA treatment induced a significant increase in FAK phosphorylation and this increase was inhibited by PP2 or Src siRNA (Fig. 8a,b). These findings were confirmed by the usages of the kinase deficient vector K298M and the constitutive active vector Y530F. Accordingly, FAK phosphorylation was inhibited by K298M, while further increased by Y530F in AGE-treated cells (Fig. 8c,d). To figure out whether FAK was phosphorylated via the formation of the Src-FAK complex, co-immunoprecipitation assay was performed. As shown in Fig. 8e, Src was associated with FAK in AGE-treated cells, not in control cells. Taken together, these data suggested that AGEs induced FAK phosphorylation by the ligation of Src and the tyrosine kinase Src.

We next investigated whether the phosphorylation of FAK played a role in the elevated endothelial permeability induced by AGEs. Cells were treated with FAK inhibitor PF573228 for 60 min before the application of AGE-BSA, and endothelial monolayer permeability was evaluated by TER and Pd. The results showed that inhibition of FAK significantly attenuated AGE-induced hyperpermeability of monolayer endothelial cells (Fig. 8f). The overall results implied that Src and FAK were important signaling molecules that mediated AGEs-induced cells. Src catalytic activity was promoted in AGE-evoked cells and functioned as a kinase for FAK, after that, phosphorylation of FAK activated the downstream pathways leading to endothelial hyperpermeability.

## Discussion

Several studies have reported that AGEs increase vascular endothelial monolayer permeability and microvascular permeability. The mechanisms underlying these responses were complex and seemed to depend on the origins of cells and organs^18^,^19^,^20^,^21^,^22^,^23^,^24^. Various proteins, including NADPH oxidase, VEGF, matrix metalloproteinases, and VE-cadherin etc, were reported to be implicated in the increased endothelial permeability in response to AGEs stimulation on different context. Our previous study showed that AGEs induced moesin phosphorylation via ROCK and p38 pathway in HMVECs^7^. In the present study, we demonstrated that in HUVECs and the primary isolated PMVECs, AGEs on the ligation of RAGE elicited a series of signaling events through the tyrosine kinase of Src, which acted like a signal node and triggered the phosphorylation of moesin, VE-cadherin, and FAK, leading to endothelial hyperpermeability. We also verified this signal mechanism in mesenteric venules *in vivo*.

Activation of Src involves dephosphorylation of Y530 and autophosphorylation of the Y419, and the phosphorylation of Src at Y419 can be used as markers for activated Src^25^. The results in this study proved that AGE application enhanced the phosphorylation of the Y419, as well as the dephosphorylation of Y530 in HUVECs in time- and dose-dependent pattern, indicating the effects of AGEs on Src activation.

In this study, AGEs were associated with RAGE to activate Src through a time- and dose-dependent phosphoarylation at Tyr419 in HUVECs, and in primary isolated PMVECs. RAGE knockout completely abated AGE-induced Src phosphorylation in PMVECs, consistent with the observation that the RAGE-specific antibody blocked the activation of Src kinase in vascular smooth muscle cells^26^. These data suggest that RAGE is required for AGE-induced Src phosphorylation, although the mechanisms by which AGEs triggered the activation of Src needed to be confirmed. Co-precipitation experiments have showed that Src interacts with both RAGE and PKCα in AGE-treated L6 skeletal muscle cells, leading to the activation of Src^27^. It is also reported that the formation of STAT5 and RAGE complex induced by dm-LDL and gly-LDL treatment enhanced Src kinase activity^28^. In primary murine aortic smooth muscle cells, mDia1 is required for RAGE-induced membrane translocation of c-Src^29^.

It is important to note that the tyrosine kinase Src works as a signaling hub where various signaling pathways diverge. Although Src can phosphorylate many proteins implicated in endothelial hyperpermeability, this study focused on the phosphorylation of moesin, VE-cadherin and FAK. Moesin is the main ERM molecule expressed in endothelium^30^,^31^, which tethering actin filaments to the plasma membrane. Moesin is maintained in an inactive state by the intramolecular interaction between the N-terminal and C-terminal domains which masks the protein–protein interaction sites. When the protein binds to the membrane lipid phosphatidylinositol 4,5-bisphosphate (PI(4,5) P2) which causes subsequent phosphorylation of a conserved C-terminal threonine, the intramolecular association is disrupted and unmasks the sites involved in the interaction with other proteins^32^. Additionally, phosphorylation of ezrin, a member of ERM family, on other sites by tyrosine kinases may also be required for specific functions^33^. The activation of moesin is engaged in regulating cellular contraction, cell motility, microvilli formation, and cell adhesion^33^. By using specific antibody against phosphor-Thr moesin, Koss *et al.* has demonstrated that TNF-α can phosphorylate threonine 558 of moesin and enhance the permeability in pulmonary microvascular endothelial cells^31^. We previously showed that AGE induced time- and dose-dependent increases in the phosphorylation of moesin at Thr^558^, which leads to the disruption of endothelial barrier^7^,^34^. The phosphorylation of moesin, F-actin disorganization and endothelial barrier dysfunction induced by AGEs were attenuated by the inhibitory mutant pcDNA3/HA-moesin^T558A^, while exaggerated by the active mutant pcDNA3/HA-moesin^T558D^, which mimicked the AGE-evoked endothelial responses^35^. In this study, activation of Src was proved to be required for moesin phosphorylation and the subsequent cytoskeleton contraction.

The intercellular adherens junctional protein, VE-cadherin, is phosphorylated, internalized and ubiquitinated in response to permeability-increasing agents such as bradykinin and histamine^17^. Study showed that VE-cadherin phosphorylation at Y658 is associated with the disruption of cell-cell junctions^36^. Inhibition of Src blocks Y658 phosphorylation of VE-cadherin and bradykinin-induced hyperpermeability^17^. In this study, the tyrosine kinase of Src is required for AGE-induced phosphorylation of VE-cadherin at Y658, and dissociation of VE-cadherin at adherent junctions (AJs), consistent with the observations that Src contributed to the phosphorylation of VE-cadherin and catenins, and to AJ disassembly^37^,^38^,^39^.

The role of FAK in the modulation of endothelial permeability has been extensively explored. Evidences show that FAK is auto-phosphorylated at tyrosine 397 after binding to integrin and provides binding sites for phosphorylation at 576, 577, 861 and 925 for several upstream signaling molecules including Src^40^. Here, we detected a Src-dependent increase in the phosphorylation of FAK at Y925. Co-immunopreciation further confirmed an interaction between FAK and the tyrosine kinase Src, which led to the phosphorylation of FAK. However, we cannot exclude Src mediated FAK phosphorylation at other epitopes, such as at Y861^41^,^42^. FAK can either strengthen or disrupt EC barrier function, which seems to depend upon the stimulus involved. Several studies have shown that FAK activation enhances endothelial barrier function in cells treated with H_2_O_2_ or thrombin^43^,^44^,^45^, while mediated the disruption of endothelial barrier in response to VEGF and transforming growth factor-β1(TGF-β1) signaling^42^,^46^. This study suggested FAK mediated AGE-induced endothelial hyperpermeability, which was in contrast to the protective role of Lyn-induced phosphorylation of FAK in stabilizing endothelial junctions.

This study did not rule out the possibility of cross-talks between moesin, VE-cadherin, and FAK signaling. For example, VEGF can promote the binding of FERM domain of FAK to the cytoplasmic tail of VE-cadherin, which facilitates the phosphorylation of beta-catenin at tyrosine-142 (Y142) and subsequently leads to the dissociation of VE-cadherin-beta-catenin complex and the breakdown of EC junctions^46^,^47^. Moreover, FAK can downregulate RhoA activity which mediates the phosphorylation of moesin^7^,^35^. Therefore, the interplay between downstream signaling of Src may also contribute to the disruption of endothelial barrier function. Last but not the least, in this paper, we just focused on the role of Src on phosphorylating para-endothelial-associated proteins implicated in endothelial hyperpermeability including the cytoskeleton contraction-associated moesin, adhesion junction-associated VE-cadherin and cell-matrix-associated FAK but not on its role on trans-endothelial-associated proteins such as caveolin-1, which was reported to be regulated by Src and participated in PMN-induced pulmonary vascular hyper-permeability^48^. Further study on caveolin-1 should be explored to come to a much more thorough conclusion in our following research.

Taken together, this study demonstrated an involvement of Src in AGE-induced endothelial hyperpermeability. Since Src localized at the node which linked upstream AGEs/RAGE signaling and the three downstream pathways, VE-cadherin, moesin and FAK, one can assume that Src may be an appropriate target for the prevention and therapy of AGEs associated microvasculopathy.

## Methods

### Cells, plasmid, antibodies and reagents

HUVECs were obtained from Sciencell. The sequences of oligonucleotide for c-Src and control siRNA were synthesized by GenePharma (Shanghai, China). The siRNA-targeted sequences were as follows: control siRNA, AATTCTCCGAACGTGTCACGT; Src siRNA, TGTTCGGAGGCTTCAACTCCT. The Src overexpressing plasmid (pcDNA3/flag-Src) and the mutants at Lys298 (kinase deficiency flag-Src-K298M, K298M) and at Tyr530 (constitutive active flag-Src-Y530F, Y530F) were synthesized by Genechem company (Shanghai, China). Antibody against Src (#2108, CST, MA, USA), p-SrcY416 (#2101, CST, MA, USA), p-moesin Thr558 (sc-12895, Santa, CA, USA), moesin (sc-136268, Santa CA, USA), p-VE-cadherin Y658 (ab27775, abcam, MA, USA), VE-cadherin (ab33168, abcam, MA, USA), p-FAK Y925(#3284, CST, MA, USA), and FAK(#3285, CST, MA, USA) were used. Src inhibitor PP2 (Cat# 529573) was acquired from Merck (Darmstadt, Germany); FAK inhibitor PF573228 (PZ0117) was purchased from Sigma (Shanghai, China).

### TER measurement

TER of HUVECs monolayer was measured using EVOM^2^ (World Precision Instruments, USA) as reported^49^. Briefly, 100 μL of cells at 10^5^/mL were seeded onto upper chamber of a trans-well with pore size of 0.4 μm. When endothelial monolayer grew confluent, TER was measured. The mean value of TER was expressed in the common unit (Ω cm^2^) after subtraction of the value of a blank cell-free filter. The relative changes of TER to baseline value were calculated by the formula:

TER = TER of experimental wells/baseline TER of experimental wells - 1.

### Dextran transendothelial flux

Cells were grown onto transwell membrane till confluent and the tracer FITC-labeled dextran (1 mg/mL) was added to the top chamber for 45 minutes. Then the concentration of dextran in upper bottom chamber was determined with a HTS 7000 microplate reader. The permeability of endothelial monolayer were evaluated by the permeability coefficient of dextran calculated as follows: Pd = [A]/t × 1/A × V/[L], where [A] is the dextran concentration in bottom chamber, t refers to time in seconds, A indicates the area of the membrane (in cm^2^), V is the volume of the bottom chamber, [L] is the dextran concentration in upper chamber.

### Preparation of AGE-BSA

AGE-BSA was prepared according to the protocol of Hou *et al.*^50^. Briefly, 0.07 g bovine serum albumin in PBS was incubated with 0.79268 g D-glucose at 37 °C for 8 weeks. Control albumin was incubated without glucose. Endotoxin was removed by using Pierce endotoxin removing gel and was less than 500 U/L.

### Western blotting

Total proteins were prepared using the lysis buffer (20 mmol/L Tris pH7.4, 2.5 mmol/L EDTA, 1% Triton X-100, 1% deoxycholic acid, 0.1% SDS, 100 mmol/L NaCl, 10 mmol/L NaF, 1 mmol/L Na_3_VO_4_) supplemented with protease and phosphatase inhibitors. Protein samples were subjected to SDS-PAGE separation, and then transferred to polyvinylidenedifluoride (PVDF) membranes. After blocked with 5% BSA, the membranes were incubated respectively with primary antibody for Src, p-Src, moesin, p-moesin, VE-cadherin, p-VE, FAK, or p-FAK at 1:1000 at 4 °C overnight, washed three times with each time for 10 min, and incubated with respective secondary antibody at room temperature for 1 h. After washed three times for 10 min each time, protein bands were visualized with chemiluminescence and densitometric analysis was operated by an imaging station.

### Transfection of HUVECs with Src siRNA and plasmid

When HUVECs cultured in DMEM/F12 without antibiotics grew to 30%–50% confluence, they were transfected with siRNA (20 nmol/L) using siRNA-Mate^TM^ according to the protocol provided by GenePharma (Shanghai, China). 48 h to 72 h after transfection, cells were treated with or without AGEs for 1 h. Then cells were lysed and subjected to Western blot.

The transfection of plasmid was carried out by LipoFilter^TM^kit. HUVECs of 70%–80% confluence were transfected with plasmids using LipoFilter^TM^ Liposomal Transfection Reagent acquired from HANBIO (Shanghai, China). Briefly, for a 6-well format, 0.4 μg DNA and 12 μl LipoFilter^TM^ was incubated in 250 μl DMEM. The DNA complexes were added to the plates and incubated for 48 h–72 h, followed by stimulation with AGEs.

### Immunofluorescent test

ECs were plated in microwells and cultured to confluence. Treated with indicated stimulation, sequentially, cells were washed in PBS three times with each time for 2 min, fixed in 4% formaldehyde for 10 min and permeabilized with 0.5% Triton X-100 at 4 °C for 15 min. Cells were washed three times, blocked in 5% BSA for 1 h, and incubated with VE-cadherin (1:200) at 4 °C overnight. After washed in PBS, cells were stained with FITC-conjugated secondary antibody (1:50). For F-actin staining, cells were incubated with rhodamine-phalloidin (2000 U/L) at room temperature for 1 h. Afterwards, cells were washed three times in PBS and detected by a Zeiss LSM780 laser confocal scanning microscope (Zeiss, Germany).

### Animal and AGE-BSA treatment of mice

The 18–20 gram male C57 mice used were purchased from the Laboratory Animal Center of Southern Medical University. RAGE knockout mice were kindly provided by Kanazawa University, Japan. The protocol using mice was approved by the Animal Care Committee of the Southern Medical University of China and in strict accordance with the Guide for the Care and Use of Laboratory Animals of the National Institutes of Health. Mice were injected intraperitoneally with BSA or AGE-BSA at 10 mg/kg for consecutive seven days. For PP2 treatment group, PP2 (2 mg/kg) was given intraperitoneally 20 min before the daily AGE-BSA injection, and for control group, equal volume of DMSO was administrated. For the assay of microvascular exudation of FITC-dextran, mice were anesthetized with an intramuscular injection of 13.3% ethyl carbamate plus 0.5% chloralose (0.65 ml/kg) before all surgical manipulations and their carotid veins were cannulated. Sequentially, mice were placed on a Plexiglas platform mounted to an intravital upright microscope and a midline laparotomy was performed, then the mesenteric venules were selected for measurement. The FITC-dextran tracer was administrated via carotid veins at 100 mg/kg in 1 mL of APSS solution, followed by continuous infusion of dextran at 0.15 mg/kg/min to maintain its concentration. The excitation wave used to detect fluorescence was 488 nm and the emission wave was 525 nm. Changes in integrated optical intensity were calculated as follows: ΔI = 1 − (Ii − Io)/Ii, where ΔI indicates the changes in light intensity, Ii is the light intensity inside the vessel, while Io is the light intensity outside the vessel.

### Isolation of mouse pulmonary microvascular endothelial cells (PMVECs)

Primary PMVECs were isolated using CD31 MicroBeads mouse according to the manufacture’s protocols provided by Miltenyi Biotec (Bergisch Gladbach, Germany). Briefly, lungs from wild type and RAGE knockout mice were excised, sliced, digested in collagenase type I, and then filtered through 70 μm nylon filters. Centrifuge cell suspension at 300 × g for 10 minutes. Move out supernatant and add 10 μl of CD31 microbeads per 10^7^ total cells. Mix well and incubate for 15 min in the refrigerator (2 °C–8 °C). Place column in the magnetic field of a suitable MACS Separator. Apply cell suspension onto the column and collect unlabeled cells that pass through the column and repeat I for several times. Finally, remove column from the separator and place it on a suitable collection tube, flushing out the magnetically labeled cells into it. Purity of mouse PMVECs were evaluated by factor VIII labeling.

### Statistics analysis

All data were expressed as means ± SE from more than three independent experiments and analyzed using SPSS 16.0 software. Statistical comparisons were performed using one-way ANOVA and P < 0.05 was considered statistically significant.

## Additional Information

**How to cite this article**: Zhang, W. *et al.* Role of Src in Vascular Hyperpermeability Induced by Advanced Glycation End Products. *Sci. Rep.* **5**, 14090; doi: 10.1038/srep14090 (2015).
